# Knowledge and Attitude Toward Shared Decision‐Making (SDM) Among Senior Students and Residents of Dental College at Riyadh City: A Cross‐Sectional Study

**DOI:** 10.1155/ijod/1543236

**Published:** 2026-04-22

**Authors:** Kiran Iyer, Fulwah Alfawzan, Rahaf Mahnashi, Mozon Alshahrani, Alanoud Alshami, Ahmed Bin Obaid

**Affiliations:** ^1^ Department of Preventive Dental Sciences, College of Dentistry, King Saud bin Abdulaziz University for Health Sciences, Riyadh, 11426, Saudi Arabia, ksau-hs.edu.sa; ^2^ King Abdullah International Medical Research Centre, Ministry of National Guard Health Affairs, Riyadh, 11481, Saudi Arabia, ngha.med.sa; ^3^ College of Dentistry, King Saud Bin Abdulaziz University for Health Sciences, Riyadh, 11426, Saudi Arabia, ksau-hs.edu.sa; ^4^ Restorative and Prosthetic Dental Sciences Department, College of Dentistry, King Saud bin Abdulaziz University for Health Sciences, Riyadh, 11426, Saudi Arabia, ksau-hs.edu.sa

**Keywords:** clinical decision-making, dental students, dentistry, informed consent, shared decision-making

## Abstract

**Background:**

Shared decision‐making (SDM) involves clinicians and patients coming to a consensus on the best course of treatment, based on the patient’s choice, after detailed deliberation on treatment options and patient priorities. Academic researchers in the region are yet to evaluate the concept of SDM in the field of dentistry; therefore, the present study aimed to assess the knowledge and attitudes of senior dental students and residents toward SDM.

**Methods:**

A cross‐sectional, self‐administered questionnaire‐based study was conducted from August 2024 to February 2025, among 176 senior dental students at a dental college at Riyadh.

**Results:**

The mean age of the senior dental students was 24.17 (± 2.13) years. Six out of seven attitude‐based questions showed significant results based on respondent’s educational levels. Students from 3^rd^ Year (D3) (OR 13.00, *p* = 0.00, CI 2.21–74.30) and 4^th^ (D4) (OR 1.17, *p* = 0.02, CI 1.17–14.50) were more likely to respond that “patients should trust clinicians to make all decisions on their behalf.” On similar lines, D3 (OR 4.20, *p* = 0.05, CI 1.98–17.90) students were more likely to feel that “shared decision‐making may cause patients to question their clinical expertise” compared to interns and residents.

**Conclusions:**

Knowledge related to SDM was found to be less than satisfactory among all the senior dental students enrolled in the study. Level of education had significant association with attitude toward SDM, those with less experience were more likely to respond that it could be questioning their expertise and was time‐consuming.

## 1. Introduction

Shared decision‐making (SDM) is an ethical imperative process where patients have the right to make decisions about their health care, for which clinicians should share information with the patient regarding treatment options, preferred management, and the likely benefits, harms, uncertainties, and risks of interventions relying on evidence‐based information [1–3].

Medical and dental services have predominantly adopted a paternalistic approach to delivering care. The basis for paternalistic care lies in the “doctor knows best” attitude, with patient’s involvement relegated to a passive role, especially concerning decisions related to patient’s own health and treatment. It is mostly viewed that doctors are best placed to decide based on their knowledge and experience on the health issue of concern, and hence solely devise patient’s medical/dental care [4].

Patients are experts on the quality of life experienced by them due to underlying oral health conditions, their choices are influenced by personal values and preferences related to oral health, and other social determinants that may influence treatment outcomes [5–7]. Professionals who are experts in diagnosis and treatment should encourage the participation of patients by providing information that will facilitate meaningful discussion. These patient values and preferences eventually reflect upon patient‐reported outcome measures (PROMs) and patient‐reported experience measures (PREMs) [8, 9].

Integrating SDM has been demonstrated to positively influence patient knowledge and satisfaction with dental services rendered, as well as a reduction in posttreatment noncompliance [9–11]. Although beneficial and widely advocated, many current dental health‐care providers have not yet fully emphasized inculcating SDM in practice, and dental education institutions are yet to integrate it into their curriculum [3].

Health‐care professional’s attitudes have been found to influence their compliance with SDM. Some health professionals believe it threatens professional autonomy, causes uncertainty, emphasizes patient autonomy to the exclusion of professional autonomy, and puts undue pressure on professionals to deliver treatment that would not be clinically appropriate [11–13]. SDM is often misunderstood as informed consent and is viewed as a barrier because it is considered time‐consuming. The literature on the topic also suggests that dental graduate’s lack of communication skills and dentist’s unawareness of evidence‐based SDM are reasons for noncompliance with SDM [14–16].

Elwyn’s three‐talk model of SDM was proposed which categorized SDM into “team talk,” “option talk,” and “decision talk,” to depict a process of collaboration and deliberation. In the year 2017, constructive critiques to this model were invited through a study; consequently, the revised model incorporated the active listening and deliberation components, and the graphical display of model helps clinician to adopt the three‐talk SDM with ease [4].

As the concept of SDM is still evolving, many countries are exploring the ways of integrating this into their health system and health professional’s curriculum. The present study is first to be undertaken among undergraduates, interns, and residents on knowledge and attitude regarding SDM in Saudi Arabia. It is important to evaluate this aspect as it will help generate baseline data about knowledge and attitude related to SDM in the region and further help establish appropriate policy and curriculum in the region. Hence, the aim of the present study was to assess the knowledge and attitude of senior dental students and residents toward SDM.

## 2. Materials and Methods

The study methodology followed the STROBE guidelines for reporting cross‐sectional studies. The methodological subsection categorization was adapted from the framework used in one of our previously published studies [17].

### 2.1. Study Design

A descriptive, cross‐sectional study was conducted to assess the knowledge and attitude toward SDM among senior students and residents of a dental college (College of Dentistry, King Saud bin Abdulaziz University for Health Sciences, Riyadh, Saudi Arabia).

### 2.2. Study Setting

This study was conducted from August 2024 to February 2025 (6 months). Prior to data collection, requisite Institutional Review Board (IRB) approval (IRB/0451/23) from King Abdullah International Medical Research Center (KAIMRC) for study protocol was obtained, permission from the concerned authorities in research unit of College Dentistry was also obtained prior to dissemination of the self‐administered questionnaire, and informed consent from all students and residents willing to be enrolled was collected.

### 2.3. Participants

Study participants were recruited using a nonprobability, purposive sampling technique from the dental college clinics. The study included all senior dental students in the 3^rd^ (D3) and 4^th^ (D4) years of undergraduate program, as well as interns and residents. These groups of students were selected as they are actively involved in clinical training and have direct interaction with patients at the dental clinics. The sample size was estimated using the level of precision formula for sample size estimation, with a proportion in the population of 27% [8] based on previous literature, a 95% confidence interval, and a power of 90% for the study. Sample size estimation was performed using G Power software (version 3.1.9.4).

### 2.4. Questionnaire

The questionnaire was adopted from two previous research papers [2, 18] on the topic with minor modifications within the demographic details. Data were collected using Google Forms, which were administered to students through email with weekly reminders. Before data collection, the questionnaire was assessed for content validity according to Lynn [19]. The scale universal agreement (S‐UA) for the questionnaire was 0.86, considered optimal. The reliability of the questionnaire was assessed through test–retest analysis, for which a total of nine senior dental students (5% of the total sample) were approached and formed the pilot sample. The responses from pilot survey was not part of final analysis. The intraclass correlation (ICC) value of 0.82 exhibited a good reliability. The questionnaire consisted broadly of two sections (knowledge and attitude) in addition to demographic details. The demographic information formed the exploratory variables, while the items assessed under the knowledge and attitude component formed the dependent variables, which were eventually evaluated against the exploratory variables. The knowledge and attitude components consisted of seven items each; responses to attitude items were based on the Likert scale ranging from 1 (strongly disagree) to 5 (strongly agree). In contrast, the knowledge component was assessed using a combination of options, which included (yes/no/not sure).

### 2.5. Statistical Analysis

Initially, the data were entered into Microsoft Excel (Microsoft Corp., New York, NY, USA) for data cleaning and coding and were later transferred into the Statistical Package for the Social Sciences (SPSS) statistical software (Version 20, 2011; IBM Corp., Armonk, NY, USA) for analysis; descriptive statistics were used to describe all the qualitative variables based on frequency and percentage. Pearson’s chi‐square analysis was used to find significant difference in knowledge and attitude with exploratory variables. Significant variables based on chi‐square analysis were explored further with multinomial regression analysis.

## 3. Results

A total of 176 students responded to the questionnaire. The mean age of the participants in the study was 24.17 (± 2.13) years. The response rate was higher among the females 99 (56.25%) than the male 77 (43.75%) counterpart. Among the responds, 46 (26.14%) of them belonged to D3, 57 (32.39%) were D4, 38 (21.59%) were interns, and 35 (19.88%) were residents. A high frequency of students 164 (93.18%) had grade point average (GPA) of 4–5, 11 (6.26%) had GPA of 3–9.3, and only one student (0.56%) had GPA of 2–2.9 (Table 1).

**Table 1:** Demographic variables in the study represented as mean, standard deviation, frequency, and percentage.

Demographic variable		Total (*N*)	Mean	Standard deviation (±)	Frequency (*n*)	Percentage (%)
Age of participants		176	24.17	2.13	—	—
Education level	D3 (3rd year)	176	—	—	46	26.14
	D4 (4th year)				57	32.39
	Intern				38	21.59
	Residents				35	19.88
Gender	Male	176	—	—	77	43.75
	Female				99	56.25
Grade point average (GPA)	2–2.9	176	—	—	1	0.56
	3–3.9				11	6.26
	4–5				164	93.18

In the knowledge component, GPA had a significant impact (*p* = 0.04) on the way students responded to one of the questions, “The majority of the patients do not want to engage in shared decision making.” Those with higher GPA scores (4–5) responded as “Yes” (25%) and “Not Sure” (15%) to the question mentioned above. As the education level of the students increased, they were significantly (*p* = 0.04) more likely to mention that interventions related to SDM have a variable effect on the treatment option. There was no significant role or difference in the way both genders replied to knowledge‐based questions of SDM (Table 2).

**Table 2:** Response of senior dental students and residents toward shared decision‐making questions assessing their knowledge.

Question (knowledge based)	Variable	*n* (%)			Total	df	Chi‐ square value (*p* value)
The majority of patients do not want to engage in shared decision‐making	Grade point average (GPA)	Yes	No	Not sure	—	4	0.04 ^∗^
	2–2.9	0 (0)	0 (0)	1 (100)	1 (100)		
	3–3.9	1 (9)	10 (91)	0 (0)	11 (100)		
	4–5	40 (25)	99 (60)	25 (15)	164 (100)		
Shared decision‐making interventions have a variable effect on the treatment option	Educational level	Yes	No	Not sure	—	6	0.04 ^∗^
	D 3	35 (76)	5 (11)	6 (13)	46 (100)		
	D 4	53 (93)	3 (5)	1 (2)	57 (100)		
	Interns	32 (84)	5 (13)	1 (3)	38 (100)		
	Residents	32 (91)	1 (3)	2 (6)	35 (100)		
Most people will understand natural frequency (e.g., 1 in every 100 people) better than a percentage	Educational level	Yes	No	Not sure	—	6	0.09
	D 3	21 (46)	7 (15)	18 (39)	46 (100)		
	D 4	32 (60)	12 (23)	9 (17)	53 (100)		
	Interns	19 (50)	3 (8)	16 (42)	38 (100)		
	Residents	15 (43)	11 (31)	9 (26)	35 (100)		

*Note: n* (frequency), df (degree of freedom), and all percentage are represented as round number.

^∗^Significance‐*p* value (<0.05).

Six out of seven attitude‐based questions showed significant results based on respondent’s educational levels. There was no significant relationship between gender and GPA, or between these factors and the attitudinal questions. All six significant questions (*p*  < 0.05) based on chi‐square analysis were further analyzed using multinomial regression (Table 3).

**Table 3:** Response of senior dental students and residents toward SDM questions assessing their attitude.

Question (attitude‐based)	Educational level (variable)	Strongly agree	Somewhat agree	Neither agree nor disagree	Somewhat disagree	Strongly disagree	Total	df	Chi‐Square
Informed consent and SDM mean the same thing	D3	5 (11)	12 (26)	5 (11)	15 (33)	9 (19)	46 (100)	12	0.01 ^∗^
	D4	2 (3)	14 (25)	8 (14)	8 (14)	25 (44)	57 (100)		
	Interns	2 (5)	6 (16)	0 (0)	6 (16)	24 (63)	38 (100)		
	Residents	2 (6)	5 (14)	3 (9)	13 (37)	12 (34)	35 (100)		
Patients should trust clinicians to make all decisions on their behalf	D3	1 (2)	13 (28)	13 (28)	12 (26)	7 (16)	46 (100)	12	0.01 ^∗^
	D4	2 (4)	16 (28)	22 (39)	11 (19)	6 (10)	57 (100)		
	Interns	2 (5)	2 (5)	10 (26)	9 (24)	15 (40)	38 (100)		
	Residents	1 (3)	9 (26)	2 (6)	9 (26)	14 (39)	35 (100)		
SDM may cause patients to question my clinical expertise	D3	4 (9)	9 (20)	14 (30)	11 (24)	8 (17)	46 (100)	12	0.02 ^∗^
	D4	4 (7)	14 (25)	17 (30)	15 (27)	6 (11)	57 (100)		
	Interns	1 (3)	5 (13)	7 (18)	11 (29)	14 (37)	38 (100)		
	Residents	0 (0)	5 (14)	4 (11)	17 (49)	9 (26)	35 (100)		
SDM is challenging because patients ask me to decide for them	D3	13 (28)	15 (33)	10 (22)	6 (13)	2 (4)	46 (100)	12	0.01 ^∗^
	D4	9 (16)	25 (44)	11 (19)	7 (12)	5 (9)	57 (100)		
	Interns	3 (8)	8 (21)	7 (18)	8 (21)	12 (32)	38 (100)		
	Residents	2 (6)	12 (34)	8 (23)	7 (20)	6 (17)	35 (100)		
SDM can only be used with patients who are sufficiently educated to discuss the treatment	D3	5 (11)	18 (39)	7 (15)	9 (20)	7 (15)	46 (100)	12	0.03 ^∗^
	D4	8 (14)	15 (26)	10 (18)	16 (28)	8 (14)	57 (100)		
	Interns	4 (11)	4 (11)	2 (5)	15 (39)	13 (34)	38 (100)		
	Residents	0 (0)	11 (31)	6 (17)	9 (26)	9 (26)	35 (100)		
SDM is unrealistic because it takes too much time	D3	9 (20)	2 (4)	5 (11)	14 (30)	16 (35)	46 (100)	12	0.01 ^∗^
	D4	1 (2)	4 (7)	10 (18)	15 (26)	27 (47)	57 (100)		
	Interns	0 (0)	3 (8)	2 (5)	11 (29)	22 (58)	38 (100)		
	Residents	2 (6)	2 (6)	0 (0)	8 (23)	23 (66)	35 (100)		

*Note: n* (frequency), df (degree of freedom), and all percentage are represented as round number.

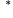
Significance‐*p* value (<0.05).

Since the residents have higher educational level, they were considered as the reference category to assess the significance among groups. D3 and D4 students had a significantly varied response for most of the attitude‐based questions.

The educational level of the dental students participating was considered the independent variable, which was assessed against their responses to the attitude‐based questions (dependent variable). Dental students from D4 (OR 0.30, *p* = 0.04, CI 0.09–0.96) and internship (OR 0.23, *p* = 0.01, CI 0.07–0.75) were less likely to disagree than the residents with the question that “SDM and informed consent mean the same.” Students at D3 (OR 13.00, *p* = 0.01, CI 2.21–74.30) and D4 (OR 1.17, *p* = 0.02, CI 1.17–14.50) were more likely to respond that “patients should trust clinicians to make all decisions on their behalf.” On similar lines, D3 (OR 4.20, *p* = 0.05, CI 1.98–17.90) students were more likely to somewhat agree that “shared decision‐making may cause patients to question their clinical expertise” (Table 4).

**Table 4:** Multinomial regression to assess significance within education level regarding attitude toward shared decision making.

Variable		Independent variable	*B*	df	Sig.	OR	95% confidence Interval for exp (*B*)	
							Lower bound	Upper bound
Informed consent and shared decision‐making mean the same thing	Somewhat disagree	D3	0.45	1	0.43	1.57	0.50	4.97
		D4	−1.17	1	0.04 ^∗^	0.30	0.09	0.96
		Interns	−1.46	1	0.01 ^∗^	0.23	0.07	0.75
		Residents	0^a^	—	—	—	—	—
Patients should trust clinicians to make all decisions on their be half	Neither agree nor disagree	D3	2.56	1	0.01 ^∗^	13.0	2.21	74.30
		D4	3.24	1	0.01 ^∗^	25.6	4.50	145.4
	Somewhat agree	D4	1.42	1	0.02 ^∗^	1.17	1.17	14.50
		Residents	0^a^	—	—	—	—	—
Shared decision‐making may cause patients to question my clinical expertise	Somewhat agree	D3	1.43	1	0.05 ^∗^	4.20	1.98	17.90
	Neither agree nor disagree	D4	1.85	1	0.01 ^∗^	6.37	1.42	28.60
		Residents	0^a^	—	—	—	—	—
Shared decision‐making is challenging because patients ask me to decide for them	Strongly agree	D3	2.97	1	0.01 ^∗^	19.50	2.19	173.40
		Residents	0^a^	—	—	—	—	—
Shared decision‐making is unrealistic because it takes too much time	Strongly agree	D3	1.86	1	0.02 ^∗^	6.40	1.20	34.01
		Residents	0^a^	—	—	—	—	—

*Note:* Last category was considered the reference group‐strongly disagree.

Abbreviation: OR, odds ratio.

^a^Reference category among the education level was residents.

^∗^ *p*  < 0.05—significant.

Further the undergraduate students at the D3 level were more like to feel that SDM is “challenging because patients could ask them to decide on their behalf” (OR 19.50, *p* = 0.01, CI 2.19–173.40) as well as is a “time‐consuming” process (OR 6.40, *p* = 0.02, CI 1.20–34.01) compared to their senior peers (residents).

## 4. Discussion

The perceived barriers to implementing SDM in the dental curricula of most countries have been access and efficiency constraints within the health‐care system; consequently, there are no clinical guidelines in this regard [20–22]. At present, students learn about SDM primarily through discussions and occasional mentions within lectures. Hence, the authors undertook the present study to assess the knowledge and attitudes of dental students toward the concept of SDM. This study represents the first of its kind in the region within the profession of dentistry.

Knowledge related to SDM had no significant difference in response, regardless of gender. This study alone has evaluated the influence of student’s GPA on their knowledge and attitudes toward SDM with patients; hence, the authors were unable to compare this variable with the existing literature on the topic.

In the present study, the GPA and educational level of the students exhibited a significant association with two questions of the knowledge component; the majority of patients do not want to engage in SDM, and SDM interventions have a variable effect on the treatment option, respectively. Interestingly, students with higher GPA scores (4–5) were more likely to feel that patients do not want to engage in SDM, which may be attributed to the “doctor knows best” approach that patients often adopt. This finding corroborates with the study by Thijs et al. [23], who analyzed patient perspectives on SDM and found that a low proportion (0.53) of the participants agreed that “the healthcare provider knows best what is good for me” was false. Most studies, such as one research by Yao et al. [22], in the Chinese population, indicated a high trust (67%) to be present among patients for the doctors, but trust plays a mediation role in patients not actively engaging in the SDM as analyzed in a study by Tian et al. [24].

As student’s progress through dental school, there is a considerable increase in their skill and decision‐making, which could influence them to feel that SDM has a variable effect on treatment options and does not always need to prompt the patient to opt for more evidence‐based treatment but instead settle for less invasive and conservative treatment options. This finding is consistent with a study conducted among dental students in United Kingdom by Sin et al. [3] who concluded that uncertainty in decisions is likely to be more of a concern to inexperienced clinicians than those with experience.

In the present study, six of the seven attitude questions related to SDM elicited a favorable response as the level of education increased. The students in the early years of their training are more likely to hold views that patients should trust clinicians to make all decisions, patients may question their clinical expertise, they may also be entrusted with decision‐making on behalf of the patient, and it would be a time‐consuming process. Our study findings are in line with other studies [11–13] which reported SDM methods application are presumed to be time‐consuming process by most respondents, except for one study conducted in the United Kingdom [3] which had a surprisingly contradictory finding that time was not a perceived barrier for SDM. Consultation time does drastically vary when SDM is put into practice at the clinics [25].

SDM emphasizes the importance of developing communication skills among students, as dental treatments are often prolonged and involve managing chronic conditions, as well as making lifelong decisions. Hence, necessitating their integration into the curriculum before students begin their clinical journey. Countries such as the United States of America have witnessed the integration of SDM into medical practice [26, 27]. In contrast, the dental curriculum has yet to incorporate it. Likewise, a consensus on integrating SDM as part of the dental curriculum is yet to be reached by the General Dental Council in the United Kingdom [11]. Academicians in China have also expressed interest in integrating SDM for the broader goal of public health, as evidenced by an international conference on this topic held in 2015 [22]. Professional education curricula such as medical education in the Middle East region of the world are highly influenced by what has been adopted and practiced in developed Western countries [28]. An article by Härter et al. [27] presents the current international perspective on SDM in developed countries worldwide.

There is a critical role of implementation science at this juncture on the topic of SDM, as barriers related to its application have been researched in almost every discipline of medical science and dentistry; its systems integration through policy is lacking [29]. Every region should consider creating its own systems implementation strategy for SDM to be fully integrated into clinical practices, as the barriers (culture, attitude, and practices) are unique to the region [30].

The results of this study must be interpreted with the following limitations in consideration. Firstly, the sample size is a concern, given that the study is cross‐sectional in nature. Also, the study had to adopt the priority sampling approach, which inherently has a smaller sample frame, and there is limited access to professionals. Given the time frame, responses were collected from students at a single institution, which may have introduced a sampling bias. There may be an inherent information bias when recalling information related to SDM, as it is not yet part of their curriculum. Variability in response based on GPA would be low, considering majority of the participants had GPA of 4 and above. In future studies, it would be interesting to analyze the knowledge and attitudes, as well as the curriculum, for SDM across the entire region (Middle East or Eastern Mediterranean).

## 5. Conclusions

Knowledge about SDM was assessed to be less than satisfactory among all the senior dental students in the present study. The experiences of these students played a pivotal role in determining their attitude toward SDM, with interns and residents exhibiting a more favorable attitude toward adopting SDM as part of their clinical practice. The introduction of the topic through mainstream teaching and learning has yet to occur for these respondents, which could explain the significantly diverse attitudes of dental students with lesser experience. A generational gap is being observed in the attitude toward engaging patients in the decision‐making process, highlighting the necessity of involving patients at all levels—mental, physical, and emotional—through effective communication and active engagement. Training students in SDM from the onset of their clinical practice can bridge this gap.

## Author Contributions

Conceptualization, methodology: Kiran Iyer, Fulwah Alfawzan, and Rahaf Mahnashi. Software: Kiran Iyer and Fulwah Alfawzan. Validation: Mozon Alshahrani and Alanoud Alshami. Formal analysis: Kiran Iyer and Rahaf Mahnashi. Investigation: Mozon Alshahrani and Alanoud Alshami. Data curation: Ahmed Bin Obaid. Writing – original draft preparation: Kiran Iyer, Fulwah Alfawzan, and Rahaf Mahnashi. Writing – review and editing: Kiran Iyer, Mozon Alshahrani, and Alanoud Alshami. Visualization: Ahmed Bin Obaid. Supervision: Kiran Iyer. Project administration: Ahmed Bin Obaid.

## Funding

No funding was received for this research.

## Disclosure

All authors have read and agreed to the published version of the manuscript.

## Ethics Statement

The study was conducted in accordance with the Declaration of Helsinki and approved by the Institutional Review Board (or Ethics Committee) of King Abdullah International Medical Research Center, Riyadh, Saudi Arabia (Protocol Code IRB/0451/23 and date of February 12, 2023).

## Consent

Informed consent was obtained from all subjects involved in the study.

## Conflicts of Interest

The authors declare no conflicts of interest.

## Data Availability

The data that support the findings of this study are openly available in FigShare at https://doi.org/10.6084/m9.figshare.30186925.
